# Potential health impact of increasing adoption of sustainable dietary practices in Sweden

**DOI:** 10.1186/s12889-021-11256-z

**Published:** 2021-07-06

**Authors:** Emma Patterson, Patricia Eustachio Colombo, James Milner, Rosemary Green, Liselotte Schäfer Elinder

**Affiliations:** 1 Department of Global Public Health, Karolinska Institutet, 171 77 Stockholm, Sweden; 2 Centre on Climate Change and Planetary Health, London School of Hygiene & Tropical Medicine, Keppel Street, London, WC1E 7HT UK; 3 Department of Public Health, Environments and Society, London School of Hygiene & Tropical Medicine, 15-17 Tavistock Place, London, WC1H 9SH UK; 4 Department of Population Health, London School of Hygiene & Tropical Medicine, Keppel Street, London, WC1E 7HT UK; 5 Centre for Epidemiology and Community Medicine, Region Stockholm, 104 31 Stockholm, Sweden

**Keywords:** Sustainability, Modelling, Health impact assessment, Adults, Non-communicable diseases, Diets, Nutrition, Climate change, Greenhouse gas emissions

## Abstract

**Background:**

An urgent transition to more sustainable diets is necessary for the improvement of human and planetary health. One way to achieve this is for sustainable practices to become mainstream. We estimated the potential health impact of wider adoption of dietary practices deemed by consumers, researchers and stakeholders in Sweden to be niche, sustainable and with the potential to be scaled up.

**Methods:**

A life table method was used to estimate the impact - changes in years of life lost (YLL) - over periods of 20 and 30 years in the Swedish population had the practices been adopted in 2010–11, when the last national adult dietary survey was conducted. The practices modelled were reducing red and processed meat (by 25, 50 and 100%), and assuming, for each stage, replacement by an equal weight of poultry/fish and vegetables +/− legumes; reducing milk intake (by 25, 50 and 100%); and reducing sugar-sweetened beverage intake (by 25, 50 and 100%). Using population data together with data on cause-specific mortality and relative risks for diet-disease outcomes, impacts were estimated for each scenario separately and in combination, for the outcomes ischaemic heart disease (IHD), ischaemic stroke, diabetes type 2 and colorectal cancer.

**Results:**

For a “moderate” combination of scenarios (changes at the 50% level), reductions of 513,200 YLL (lower-upper uncertainty estimate 59,400-797,900) could have been achieved over 20 years and 1,148,500 YLL (135,900-1,786,600) over 30 years. The majority (over 90%) of YLLs prevented were related to IHD, and the majority were in men. The singular practice that had the most impact was reducing the intake of red and processed meat and replacing it with a mixture of vegetables and legumes. Reducing milk intake resulted in an increase in YLL, but this was compensated for by other scenarios.

**Conclusion:**

If these practices were more widely adopted, they would be expected to lead to improvements in public health in Sweden. Over the long term, this would translate to many premature deaths postponed or prevented from a number of chronic diseases, to the benefit of individuals, society, the climate and the economy.

**Supplementary Information:**

The online version contains supplementary material available at 10.1186/s12889-021-11256-z.

## Background

An unhealthy dietary pattern is one of the largest contributors to poor health [1]. The way food is produced, distributed and consumed globally also contributes to about 25–30% of total greenhouse gas emissions (GHGE) [2], as well as impacting other aspects of environmental sustainability [3]. Changing the diet therefore has the potential to both improve public health and contribute to reductions in GHGE [4]. If the internationally agreed sustainability targets, of which the Paris Agreement and the UN Sustainable Development Goals are the most high profile, are to be met, substantial changes to current diets will be required, particularly in industrialised, wealthy nations [5]. Although the Swedish Food Agency was one of the first to produce food-based dietary guidelines that considered environmental sustainability as well as health [6], for average Swedish diets to be in line with e.g. the Eat-Lancet Planetary Health Diet [4] would require a considerable increase in vegetable, fruits, whole grains, legumes and nut intakes, and less red meat, processed meat, added sugar, refined grains, and starchy vegetables.

Achieving behaviour change is challenging, and so an urgent question is how best to achieve the major shifts required. The current report is part of a 4-year research programme “Mistra Sustainable Consumption – from niche to mainstream” financed by the Swedish research council Mistra. The programme aims to contribute to the transition to sustainable consumption by generating knowledge on how “niche” sustainable practices, already in place, can become mainstream in Sweden in the areas of food, vacation and home furnishings [7]. In a previous stage of the programme, a wide range of public and private stakeholders identified a number of dietary practices as being currently niche, sustainable and suitable for scaling up. The definition of sustainability used was broad, but the focus of this analysis is on those practices expected to both benefit health and reduce climate impact.

The increasing adoption of practices can be framed in terms of Rogers’ Diffusion of Innovation Theory, where a population can be divided into five different segments based on their propensity to adopt a specific innovation: innovators, early adopters, early majorities, late majorities and laggards [8]. “Niche” practices can be thought of as those of innovators and early adopters, who can be considered motivated to embrace innovations immediately and without further incentives. In contrast, the early and late majority require more persuasion and/or support in order to change their behaviour, as, until a new norm is reached, powerful negative societal and commercial influences can easily overwhelm the individual consumer’s efforts to take action [9]. There are many ways to encourage changes in values, norms and practices at a population level, ranging from information to consumers, to more upstream solutions, such as economic instruments (i.e. subventions/taxes) and regulation. A recent report concluded that in order to achieve the considerable dietary changes necessary to reduce Sweden’s GHGE from food - on average 1.5 ton per year for women and 2.0 ton per year for men [10] – upstream solutions such as incentives or taxes, rather than just information, are necessary [11]. Such policies are however politically sensitive, so decision makers are often reluctant to use these strategies. For policymakers to take such steps, estimates of the potential health and environmental gains resulting from improvements to diet need to be robustly and consistently demonstrated, and health impact modelling is one way of doing this.

This study aims to estimate the long-term (20- and 30-year) public health impacts of adopting the food-related practices identified by the stakeholders referred to above for which health impact data is available.

## Methods

### Identification of scenarios

A previous work package of the main “Mistra Sustainable consumption” programme gathered wide-ranging examples of what were perceived to be niche sustainable practices related to food production and consumption, with potential for scaling up [12]. Briefly, suggestions were solicited via workshops with representatives from the programme’s 20-odd stakeholder partners and a similar number of researchers, by literature reviews and studying reports, websites, magazines and social media in relation to sustainable consumption practices in Sweden and abroad. They were also gathered using a web-based questionnaire, disseminated in fora for people interested in sustainable consumption between April and October 2018, and interviews with international researchers. Participants were not intended to be representative, and no attempt was made to define sustainability, so suggestions were made from the point of view of climate impact, biodiversity, human health, social impact, animal welfare, community resilience etc. The full list of practices was then compiled [12], taking no account of their *effectiveness* to reduce GHGE (this is the focus of other sections of the research programme). From all of the food-related practices suggested, we identified the ones which would plausibly result in improved health as well as lower GHGEs. These were the practices that had been labeled as: 1) “Choose meat with lower climate impact instead of red meat”, 2) “Swap animal-based products for vegetable-based alternatives” and 3) “Reduce ‘unhealthy’ consumption” [12]. These are practices broadly in line with current national [13], Nordic [14] and international [15] dietary guidelines, as well as with e.g. the Eat-Lancet Commission report [4], and therefore expected to also have a lower climate impact.

In order to conduct the health impact modelling we first operationalised these practices as more specific scenarios, taking into consideration dietary factors for which robust data on potential health impacts was available, as defined by the Global Burden of Disease (GBD) 2017 analysis [16]. In the end we modelled reductions in red and processed meat and assumed replacement by a) poultry/fish, b) vegetables, and c) a 50:50 mixture of vegetables and legumes. Another animal-based product included in the GBD is milk so we modelled reductions in intake, assuming replacement by a plant-based drink. The practice “Reduce ‘unhealthy ‘consumption” referred to intake of foods with low nutritive value; the most closely related factor in GBD was sugar-sweetened beverages (SSBs) so this was chosen, even if GHGEs associated with SSBs are relatively low. All replacements were by equal weight. For each scenario we modelled partial (25 and 50% decrease with replacement) and full (100% decrease with replacement) implementation in order to illustrate the range of potential impact. In order to be able to summarise the overall potential impact, we also grouped the changes into combinations. These we termed “minor” (changes at the 25% level), “moderate” (the 50% level) or “extensive” (the 100% level). Changes were modelled at the level of food groups rather than individual foods. For some of the assumed compensations it was not possible to model what effect the substitutions would have on health, as these are not considered risk factors according to GBD 2017. As such, in our models, replacements by poultry, fish or plant-based milk were neutral in terms of the health impact.

### Baseline consumption data

Estimates of baseline dietary intake in the adult Swedish population were from the latest nationally representative adult dietary survey, Riksmaten 2010–11, conducted by the Swedish Food Agency. The data is publicly available in fully anonymised form [17]. Briefly, 1797 adults aged 18–80 recorded all food and drinks consumed for four consecutive days in a web-based diary, between May 2010 and July 2011. The participants were reasonably representative of the general population, apart from slightly higher education level among participants than non-participants. The method used has been validated with respect to both energy intake and biomarkers [18, 19]. From this, the average intakes of red meat, processed meat, vegetables, legumes, milk and SSBs were calculated. Definitions of dietary factors used in the GBD 2017 were followed [16], namely red meat included beef, pork, lamb and other meats but not poultry or processed meat; processed meat is any meat that has been smoked/cured/salted/chemically preserved; vegetables excluded pickled vegetables, starchy vegetables and legumes; SSBs have ≥50 kcal/226.8 ml and excluded juices. Information on ingredients in mixed dishes was also available, allowing for detailed extraction of the dietary factors by summing components consumed both as whole foods and as ingredients. Intakes were calculated for men and women separately. As all data from the dietary survey has been anonymised this study involved no personal data; ethical permission was not required for this analysis according to Swedish law [20].

### Associations between dietary factors and disease risk

Relative risks (RR) for associations between dietary factors and four disease outcomes were taken from GBD 2017. For the dietary factors identified, there is evidence, of at least moderate strength, for an association with ischaemic heart disease (IHD), ischaemic stroke, type 2 diabetes and colorectal cancer [21]. In addition, these diseases accounted for 19.9, 5.0, 1.6 and 3.4% of total deaths in Sweden respectively in 2017 [1] and are, together with lung cancer, the diseases with a dietary risk factor that account for the most deaths. As such they are also of public health importance. RRs which are expressed in the GBD study in terms of a harmful risk factor (e.g. “diet low in vegetables”) were inverted to create RRs for a positive change in diet. RR in GBD 2017 are modelled and presented per 5-year age intervals, from 25 years and upwards, for a given unit of dietary change, e.g. 100 g increase in vegetable intake. Rates for 15–24 years were assumed to be the same as for 25–30 and a single RR for the entire population was then calculated by weighting the RRs for each 5-year age interval according to the population structure and taking the average.

### Population data

As the latest national dietary data for Swedish adults is from 2010 to 11 we used 2011 as the baseline year for our analysis. Data on population size for 2011 was obtained from the national statistics agency, Statistics Sweden [22] in 1-year age intervals. This was summed to 5-year age intervals and for each interval a sex-specific (weighted) mean age was calculated. Data on all-cause mortality for each age in 2011 was obtained from the same source and neonatal deaths were calculated and excluded. Total deaths were summed to 5-year age intervals for each sex.

Disease-specific mortality rates for Sweden in 2011 were taken from the GBD 2017 database [23]. Data was taken from GBD 2017 rather than directly from the national source, as the grouping of international classification of disease (ICD) cause of death codes made publicly available by the National Board of Health and Welfare do not always overlap perfectly with the groupings used in GBD 2017. Doing so ensured that all deaths for the same ICD codes that the RR are based on were included. Death rates in GBD 2017 are presented for 5-year age intervals. Using the weighted mean age of the Swedish population in each interval, the rate for each 1-year age interval was interpolated using one-way spline interpolation using the programme SRS splines, available as an add-in to Excel [24]. Based on the population size for each 1-year age interval, the number of deaths at each age for each disease was estimated.

### Health impact modelling

The health impact assessment was performed using the IOMLIFET life table method [25], adopted in R [26]. Life table calculations allow for the changes in future population shape that are induced by changes in mortality risks, and the subsequent changes in survival curves can be summarised as e.g. years of life lost (YLL) or changes to life expectancy [25]. YLL is a summary measure of premature mortality; it represents the years of potential life lost across a population due to premature deaths, taking into account the age at which deaths occur. Life tables were constructed for males and females separately. All data on population size, all-cause mortality and disease-specific mortality as described above were age- and sex-specific. The following assumptions were made: the diet changes are made instantly and then kept constant, and underlying mortality rates remain constant for the duration of follow-up. The exposure–response functions were assumed to be log-linear. Risks were calculated for each dietary exposure taking into account the size of the dietary change being modelled, e.g. 55 g increase in vegetable intake. In scenarios where more than one dietary exposure affected the risk for the same disease, e.g. in one where vegetables increased and processed meat reduced, the combined predicted change in risk for IHD (for which both are risk factors) was derived by multiplying the risk for each factor together.

We modelled the impact on mortality on IHD, stroke, type 2 diabetes and colorectal cancer. As these outcomes are chronic diseases, there is often a cumulative effect of an exposure; this we estimated to reach a maximum after approximately 10 years for IHD, stroke and type 2 diabetes, and 30 years for cancers [26]. Chronic diseases, particularly cancer, also have long lag times between exposure and onset of outcome, and so no change in cancer risk was assumed for the first 10 years [26]. To account for these, time-varying functions were used in the models, based on cumulative distribution functions of normally distributed variables (s-shaped curves). An uncertainty interval (UI) was constructed around each estimate based on the lower and upper ranges for the RRs. The output was changes in YLL for the population over time periods of 20 and 30 years for each outcome separately. These were then summed to arrive at the cumulative total. All analyses were conducted in R [27].

## Results

The baseline intakes of the dietary factors and the relative risks for each factor and disease outcome used are presented in Table 1. Men had a higher baseline intake of all selected dietary factors, except for vegetables.

**Table 1:** Average intakes of each dietary factor at baseline, size and direction of the unit for the relative risks, and the relative risks (RR) for each disease assumed

	Baseline intakes		Relative risks (95% CI)				
Dietary factor	Men (g/day)	Women (g/day)	Unit and direction for RR (g)	IHD^**a**^	Ischaemic stroke^**a**^	Diabetes type 2^**a**^	Colorectal cancer^**a**^
Red meat	67.7	41.9	-100	–	–	0.80 (0.97–0.68)	0.86 (0.97–0.76)
Processed meat	43.5	25.5	−50	0.56 (0.97–0.4)	–	0.59 (0.76–0.48)	0.85 (0.91–0.79)
Vegetables	155.2	169.6	+100	0.87 (0.95–0.79)	0.87 (0.97–0.79)	–	–
Legumes	12.3	11.9	+50	0.76 (0.89–0.66)	–	–	–
Milk	224.2	171.9	+226.8	–	–	–	0.90 (0.96–0.83)
SSB	110.1	72.7	−226.8	0.81 (1.05–0.66)	–	0.83 (0.91–0.76)	–

*SSB* sugar-sweetened beverages, *IHD* ischaemic heart disease

- indicates no established relationship between the dietary factor and the disease

^a^Single weighted average of GBD 2017 RRs for dietary risk factors per 5-year intervals, taking into account the Swedish population structure in 2011, and inverted to create RRs for a positive change in diet.

The results of the health impact assessment are presented in Table 2, as changes in YLL per scenario and per combination of scenarios. The results from the health impact modelling suggest that, had Swedish adults made the “moderate” combination of these dietary changes in 2011 – i.e. a 50% reduction in red and processed meat (with replacement by vegetables), in milk and in SSBs - a reduction of approximately 513,200 YLL could have been achieved over 20 years (Table 2). If the more “extensive” combination had been adopted - a 100% reduction in red and processed meat (with replacement by vegetables and legumes), in milk and in SSBs - a reduction of 1,076,900 YLL could have been achieved. Although the uncertainty ranges for the estimates were wide, reflecting the wide ranges of the RRs for many of the dietary factor-disease outcome pairs, even at the lower ranges the estimates for even the “minor” combination of scenarios (changes at the 25% level) were positive. Over 30 years, the impact was more than twice as great (Table 2).

**Table 2:** Estimates of the reductions in total years of life lost (YLL) that could be achieved over 20 and 30 years if dietary changes were made in 2011, per scenario and in combination

	Cumulative reduction in total YLL			
	Over 20 years		Over 30 years	
Scenarios modelled	Estimate	(Lower - Upper)	Estimate	(Lower - Upper)
**Replacing red & processed meat with poultry/fish**^a^				
25% less red & processed meat*	176,300	(16,100–269,700)	400,400	(39,600–613,600)
50% less red & processed meat	336,200	(31,700–501,000)	765,500	(78,300–1,144,100)
No red or processed meat	611,200	(61,600–866,900)	1,397,300	(152,200–1,991,100)
**Replacing red & processed meat with vegetables**				
25% less red & processed meat	233,600	(37,300–361,600)	527,300	(86,600–817,300)
50% less red & processed meat**	440,800	(73,800–659,900)	997,600	(171,300–1,497,900)
No red or processed meat	786,100	(144,300–1,107,300)	1,787,400	(335,000–2,529,000)
**Replacing red & processed meat with vegetables and legumes**				
25% less red & processed meat	293,900	(69,000–440,200)	661,900	(156,800–993,700)
50% less red & processed meat	544,300	(135,800–781,900)	1,230,000	(308,800–1,774,100)
No red or processed meat***	935,200	(261,800–1,248,600)	2,126,100	(596,400–2,853,900)
**Replacing milk consumption with plant-based drink**^a^				
25% less milk intake*	-7,00	(- 300 – - 1,200)	- 6,200	(- 2,400 – - 11,000)
50% less milk intake**	-1,400	(- 500 – - 2,500)	-12,500	(- 4,800 – - 22,500)
No milk intake***	-2,900	(- 1,100 – - 5,300)	-25,600	(- 9,600 – - 46,900)
**Replacing SSB consumption with water**^a^				
25% less SSB intake*	37,200	(-6,900–71,500)	82,300	(-15,200–158,200)
50% less SSB intake**	73,800	(- 13,900–140,600)	163,300	(-30,600–311,300)
No SSB intake***	144,700	(-27,900–270,600)	320,400	(-61,600–600,200)
**Combination**				
“Minor changes” (sum)*	212,800	(8,900–339,900)	476,600	(22,000–760,900)
“Moderate changes” (sum)**	513,200	(59,400–797,900)	1,148,500	(135,900–1,786,600)
“Extensive changes” (sum)***	1,076,900	(232,800–1,513,900)	2,420,900	(525,200–3,407,200)

*SSB* sugar-sweetened beverages, *YLL* years of life lost

Replacements were of equal weight. Vegetables and legumes were 50:50. Numbers rounded to nearest 100.

^a^Replacement food category was neutral in the health impact model

In general, scenarios involving lower meat intake had greater impacts than those involving milk or SSBs. Reductions in YLL were greater when red and processed meat was replaced with a combination of vegetables and legumes, rather than vegetables alone. One of the scenarios, a reduced intake of milk, resulted in an increase of YLL, as there is evidence that a higher intake of milk is associated with a reduced risk of colorectal cancer. However, other changes, such as reducing red and processed meat can compensate for this: for example the increase in YLL from a 100% reduction in milk intake (2900 YLL over 20 years) was compensated by the 50% reduction of red and processed meat (reduction of 2800 YLL over 20 years). An additional file shows this in more detail (see Additional file 1).

**Fig. 1:**
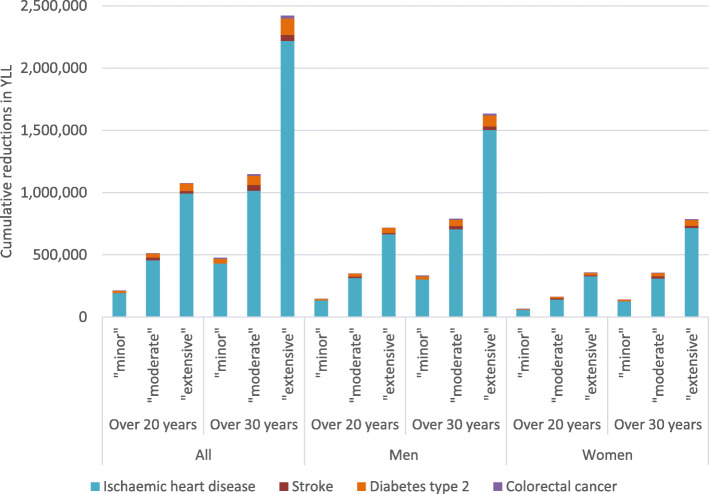
Cumulative reductions in YLL over 20 and 30 years for the different diet scenario combinations. See text/Table 2 for a description of the combinations

The results of the combinations of scenarios are presented in Fig. 1, showing the breakdown by outcome and sex. The disease outcome that was affected most by the changes was IHD mortality, which accounts for the vast majority of the reductions in total YLL (ca 90%), followed by diabetes type 2 (Fig. 1). The longer lag time for colorectal cancer means that only very small reductions in YLL would be seen after 20 years; reductions would be seen mainly after 30 years. Reductions in YLL were greater for men (Additional file 1), which was expected as the absolute changes modelled were relative to the baseline intake and intakes were generally higher among men (Table 1). Estimates for each change and disease outcome and for each sex are available in Additional file 1.

## Discussion

Wider adoption of the dietary practices identified by the “Mistra Sustainable Consumption” programme as niche, sustainable and with potential to become mainstream would be expected to result in considerable public health benefits, especially for men, in addition to a likely reduction in diet-related GHGEs. Taking 2010–11 as the starting point for implementation, we modelled what the impact in Sweden might have been in terms of deaths prevented or postponed over 20 and 30 years. The results suggest that the gains could have been in the hundreds of thousands of YLL, possibly in the region of a million for the combination of scenarios labelled “moderate” (changes at the 50% level) or “extensive” (changes at the 100% level). To put this in perspective, in Sweden in 2017, the number of YLL from all causes was approximately 1,248,000 according to GBD 2017 [21]. Approximately 16% of these were due to IHD (ca 203,000), and poor dietary habits (the cumulative effect of all 15 dietary risks included in GBD) was the biggest risk factor. As the biggest change in our dietary scenarios was for YLL due to IHD, it is easy to see how the cumulative figure over a longer time period reaches a substantial number. The practice that had the most impact was reducing the intake of red and processed meat and replacing it with a mixture of vegetables and legumes. The results suggest that this practice alone could prevent about a fifth of YLL due to IHD.

As the most recent national dietary data in Sweden is from 2010 to 11, it is worth considering how relevant the proposed scenarios are today. Changes in per capita supply data between 2010 and 2019 [28] suggest a 18% decrease in pork, a 7% decrease in beef and a 20% increase in poultry volumes. No clear trend is seen for vegetables using the same source, but the market for “meat alternatives” has increased over 15% year-on-year between 2017 and 2019 [29]. A internet panel survey of vegetarian practices suggests a similar trend between 2016 and 2019: while the proportion of adults who rarely or never ate vegetarian meals was 48% in 2019, the proportion who never ate vegetarian meals had fallen from 22 to 15% [30]. It should be noted that neither per capita supply data nor market analysis is a substitute for individual consumption data but can be useful to observe trends [31]. These would suggest that the scenario of replacing red meat (with poultry or something else) is already being practiced by some consumer segments – from “innovators” to the “early majority” [8] – and have the potential to be scaled up. In contrast, the per capita sales volume of SSBs has increased slightly since 2010 [32]. Rogers lists compatibility with existing values, norms and practices as one of five factors determining the success of an innovation. Others are 2) relative advantage (the greater the perceived relative advantage of an innovation by the user group, the more rapid its rate of adoption is likely to be); 3) simplicity and ease of use; 4) trialability (the degree to which an innovation can be experimented with on a limited basis); and 5) observable results (the easier it is for individuals to see the results of an innovation, the more likely they are to adopt it) [8]. In order to accelerate the dietary shift required, all these factors could be taken into consideration by stakeholders and decision-makers who want to bring about change.

It is always important to take account of several aspects of sustainability simultaneously so that e.g. health is not prioritised at the expense of the environment, or social sustainability. In general, the overlap between foods that have lower environmental impact and also improve health is usually high, with the notable exception of fish and sugar, where health and environmental impacts may act in the opposite directions [33]. We were not able to consider other impacts that substitutions within food groups could have in more detail. However in another part of the “Sustainable Consumption” project, the impacts of a number of potential “replacement” products relevant for these scenarios (e.g. legume-based products, plant-based drinks) have been quantified from the Swedish perspective [34], confirming their lower GHGE impact, even when production, transport from abroad, packaging, etc. is taken into account.

One of the major limitations of simulations is that replacements may have consequences for energy balance and nutritional adequacy, as what and how much replacement occurs in reality is difficult to know. We assumed here replacement by equal weight, not by energy. For example in one scenario we assumed a total reduction in processed and red meat, and compensating for this by increasing vegetables and legumes (in equal amounts) by the same weight. This increase was, on paper, not excessive and would bring average intakes of total fruits, vegetables and legumes up to 384 g for men and 396 g for women, still far below the recommended intakes of 500 g [13]. If these were consumed in their unprocessed form it would likely mean a shortfall in energy intake, due to the lower energy density of this food group. It is however likely that replacement would also involve more processed vegetable- and legume-based products, which are more energy-dense than unprocessed vegetables and legumes, as the availability of these has increased dramatically in recent years in Sweden [29]. Given half of all Swedish adults are living with the effects of a prior or ongoing positive energy balance (i.e. are overweight or obese [35]), for many energy deficits may lead to further health gains. Similarly, the impact associated with reduced SSB consumption are also possibly underestimated as further gains could be mediated through a reduction in obesity, for which high SSB consumption is a risk factor.

A more critical issue is if the foods that are reduced are important sources of nutrients that are not compensated for. Some of our modelled scenarios would be almost neutral in terms of impact on micronutrient intake, for example replacing SSB with water. Others are more complex. For example, meat is a rich source of nutrients such as iron, selenium, zinc and some B-vitamins. However, a study from the Nordic region examined this (using, for the Swedish part, the same dietary survey data as in our study) and concluded that the effects on overall dietary quality would be minimal if processed meat was reduced to zero, and if average red meat was reduced to the WCRFs 2007 population-level recommendation of 43 g per day [36]. They modelled scenarios involving both replacement by other meat, non-meat, and with or without energy compensation. Another way of looking at the replacements at food level and ensuring that the overall diet is nutritionally adequate would be to perform an optimisation analysis using linear programming [37] and this is planned in a future study.

Another limitation of simulations or models is that they remain theoretical. Indeed, the relationship between the health and environmental impacts of self-selected diets is more complex than that between single foods/food groups [38]. The modelled changes may also be less acceptable to consumers than what is assumed. Vieux et al. examined actual dietary patterns in six European countries, including Sweden, and concluded that exclusion of entire categories of food is not necessary to achieve health and climate benefits, and a “more sustainable” diet with “moderate” amounts of animal-based products is probably realistic, as it is already adopted by nearly one in five adults [38] corresponding to the population segments innovators, early adopters and some of the early majority according to the Diffusion of Innovations theory [8]. We therefore made sure to include scenarios where animal-based products were still included to a large degree, as well as being more extensively reduced.

Our results complement those of Saha et al. [39] who estimated 1-year health gains if food and nutrient intakes were in line with Nordic Nutrition Recommendations. They used the PRIME model, which is different from our method and based on other premises. They estimated that 6405 deaths in a year in Sweden would be prevented/delayed from cardiovascular diseases and diet-related cancers, or 14.4% of the total. In line with our results, the majority of YLL reductions were also from IHD. However other differences make the results difficult to compare: they used the same dietary data but based their model on population data from 2016, not 2011 which was 5.4% smaller; they modelled nutrients (fat, salt, dietary fiber, energy) and only one food group (fruits and vegetables (and not meat)). They also assumed that their health gains occurred in the same year, not allowing for a lag time as we have done, and not taking into account the impact on the population structure over time. Assuming a constant effect over 20 years or 30 years, this would correspond to 128,000 or 192,000 prevented or postponed deaths in their study. Using the same IOMLIFET approach, a scenario for the UK (population 67 million) suggested almost 7 million YLL would be saved over 30 years if diets were in line with WHO dietary recommendations [26]. A study from Italy using the IOMLIFET model that we used predicted that reducing beef by 63% (to 150 g per week) and processed meat by 80% (to 50 g per week) would reduce YLL by 9 and 20 million respectively over 30 years [40]. This is a similar range to our numbers, when Italy’s population size (about 6 times larger) is taken into account.

Other strengths and limitations are that we included all food-based dietary factors in GBD 2017 that could be connected to the proposed scenarios but did not consider nutrient-based dietary factors. Where several dietary exposures affected the same disease, the risks were multiplied together. It is possible that this leads to an over-estimation, but this is commonly done in other models too, e.g. PRIME, and due to lack of information on mediation/overlap. We only examined YLL, not years of life lived with disability (YLD), another widely used measure of health impact, which means that we have most likely underestimated total health benefits by not accounting for impacts on morbidity. We also did not consider here the effects that any reduction in body mass index (BMI) may have had, either due to negative energy balance as a result of a scenario, or as a scenario in its own right. Reducing excess consumption (and waste) is one obvious way to reduce the environmental impact of a diet, as this is determined by both the quality and quantity of food consumed [38].

The population has increased since 2011, from 9.48 million in 2011 to 10.33 million in 2019 [22], an increase of 8.9% so the reductions in YLLs may be underestimated. The population structure has remained similar: the proportion of men increased by less than 1%, life expectancy at birth increased by 1.9% for men and 1.3% for women during the last decade. Data on disease-specific death rates were taken from GBD 2017 rather than directly from the national source to ensure deaths from and RRs for diseases were for exactly the same disease codes, but the differences in deaths from both sources were minimal (< 1%). The expected benefit to the environment was limited to the effect on GHGEs, but other aspects such as water and land use are also important. Although a clear socioeconomic gradient is seen with dietary quality, the impact of dietary changes on economic sustainability was not possible to include in this analysis.

## Conclusion

The widespread adoption of dietary practices identified as being niche today but with the potential to become mainstream, could result in considerable improvements in public health in Sweden, particularly over the long term. Although modeling health impacts requires making assumptions and a level of uncertainty, this potentially translates to many premature deaths postponed or prevented from a number of chronic diseases, primarily IHD, to the benefit of the individual, society and with probable benefits for the economy and the climate. Some motivated consumer segments may be willing to adopt more sustainable practices right away. However, in order to accelerate the transition to a more sustainable diet within the timeframe that meeting commitments to international agreements regarding sustainability requires, policymakers should consider more potent strategies in order to persuade far more of the population to do so.

## Supplementary Information

**Additional file 1.** Cumulative gains in YLL over 20 or 30 years, per sex and disease outcome.

## Data Availability

The dietary survey data Riksmaten 2010–11 is publicly available from the Swedish Food Agency, https://www.livsmedelsverket.se/om-oss/psidata/apimatvanor The data on disease specific mortality rates is publicly available from GBD, http://ghdx.healthdata.org/gbd-results-tool The relative risks for diet-disease associations are publicly available from GBD 2017, http://ghdx.healthdata.org/record/ihme-data/gbd-2017-burden-risk-1990-2017 The population data is publicly available from Statistics Sweden, http://www.scb.se/be0101 Other datasets generated during the current analysis are available from the corresponding author on reasonable request.

## Abbreviations

GBD: Global Burden of Disease
GHGE: Greenhouse gas emissions
ICD: International Classification of Disease
IHD: Ischaemic heart disease
IOMLIFET: Institute of Occupational Medicine Life Table
RR: Relative risk
SSB: Sugar-sweetened beverages
YLL: Years of life lost
