# Supplementary material for: Potential health impact of increasing adoption of sustainable dietary practices in Sweden
Source: BMC Public Health. 2021 Jul 6;21:1332. doi: 10.1186/s12889-021-11256-z (PMC8261973; doi:10.1186/s12889-021-11256-z)
Supplement: Supplementary file 1 — Additional file 1. Cumulative gains in YLL over 20 or 30 years, per sex and disease outcome. [file 12889_2021_11256_MOESM1_ESM.pdf]

Table 1 Cumulative gains in YLL over 20 or 30 years, per sex and disease outcome

	Ischaemic heart disease		Stroke		Diabetes type 2		Colorectal cancer		Sum	
	Estimate	Lower-Upper	Estimate	Lower-Upper	Estimate	Lower-Upper	Estimate	Lower-Upper	Estimate	Lower-Upper
Women, cumulative gains in YLL over 20 years										
**Replacing red & processed meat with poultry/fish#**										
25% less red & processed meat*	48130	(2603 – 74714)	0	(0 – 0)	4823	(2085 – 6979)	504	(212 – 803)	53457	(4901 – 82496)
50% less red & processed meat	93346	(5198 – 142362)	0	(0 – 0)	9230	(4092 – 13087)	990	(422 – 1560)	103566	(9712 – 157009)
No red or processed meat	175013	(10323 – 257879)	0	(0 – 0)	16888	(7858 – 23051)	1905	(828 – 2938)	193806	(19008 – 283867)
**Replacing red & processed meat with vegetables**										
25% less red & processed meat	62777	(8362 – 98455)	4635	(1023 – 7787)	4823	(2085 – 6979)	504	(212 – 803)	72740	(11682 – 114023)
50% less red & processed meat**	120500	(16602 – 184455)	9143	(2034 – 15243)	9230	(4092 – 13087)	990	(422 – 1560)	139863	(23151 – 214345)
No red or processed meat	221881	(32821 – 324147)	17891	(4049 – 29386)	16888	(7858 – 23051)	1905	(828 – 2938)	258565	(45556 – 379521)
**Replacing red & processed meat with vegetables and legumes**										
25% less red & processed meat	83589	(18370 – 127005)	2317	(509 – 3907)	4823	(2085 – 6979)	504	(212 – 803)	91233	(21176 – 138693)
50% less red & processed meat	158684	(36499 – 233860)	4635	(1023 – 7787)	9230	(4092 – 13087)	990	(422 – 1560)	173540	(42036 – 256294)
No red or processed meat***	284144	(71117 – 395337)	9143	(2034 – 15243)	16888	(7858 – 23051)	1905	(828 – 2938)	312079	(81837 – 436569)
**Replacing milk consumption with plant-based drink#**										
25% less milk intake*	0	(0 – 0)	0	(0 – 0)	0	(0 – 0)	-283	(-109 – -504)	-283	(-109 – -504)
50% less milk intake**	0	(0 – 0)	0	(0 – 0)	0	(0 – 0)	-571	(-219 – -1026)	-571	(-219 – -1026)
No milk intake***	0	(0 – 0)	0	(0 – 0)	0	(0 – 0)	-1165	(-440 – -2125)	-1165	(-440 – -2125)
**Replacing SSB consumption with water#**										
25% less SSB intake*	11230	(-2623 – 21994)	0	(0 – 0)	823	(418 – 1208)	0	(0 – 0)	12053	(-2205 – 23202)
50% less SSB intake**	22303	(-5254 – 43383)	0	(0 – 0)	1634	(833 – 2389)	0	(0 – 0)	23937	(-4421 – 45772)
No SSB intake***	43926	(-10528 – 84291)	0	(0 – 0)	3215	(1651 – 4671)	0	(0 – 0)	47141	(-8877 – 88961)
* Combination "Minor changes" (sum)	59361	(-20 – 96708)	0	(0 – 0)	5646	(2503 – 8187)	221	(103 – 299)	65228	(2587 – 105194)
** Combination "Moderate changes" (sum)	142803	(11348 – 227838)	9143	(2034 – 15243)	10864	(4925 – 15477)	419	(203 – 534)	163229	(18510 – 259092)
*** Combination "Extensive changes" (sum)	328069	(60588 – 479628)	9143	(2034 – 15243)	20103	(9509 – 27721)	740	(387 – 813)	358056	(72519 – 523405)

SSB, sugar-sweetened beverages.

Replacements were of equal weight. Vegetables and legumes were 50:50. Numbers rounded to nearest 100.

#Replacement food category was neutral in the health impact model.

Table 1 (continued) Cumulative gains in YLL over 20 or 30 years, per sex and disease outcome

	Ischaemic heart disease		Stroke		Diabetes type 2		Colorectal cancer		Sum	
	**Estimate**	**Lower-Upper**	**Estimate**	**Lower-Upper**	**Estimate**	**Lower-Upper**	**Estimate**	**Lower-Upper**	**Estimate**	**Lower-Upper**
Women, cumulative gains in YLL over 30 years										
**Replacing red & processed meat with poultry/fish#**										
25% less red & processed meat*	104378	(5639 – 162149)	0	(0 – 0)	10573	(4571 – 15300)	4493	(1893 – 7156)	119444	(12102 – 184604)
50% less red & processed meat	202688	(11260 – 309547)	0	(0 – 0)	20237	(8971 – 28696)	8827	(3757 – 13905)	231752	(23988 – 352148)
No red or processed meat	380892	(22363 – 562568)	0	(0 – 0)	37033	(17228 – 50554)	16985	(7376 – 26204)	434910	(46967 – 639326)
**Replacing red & processed meat with vegetables**										
25% less red & processed meat	136197	(18113 – 213814)	9915	(2187 – 16657)	10573	(4571 – 15300)	4493	(1893 – 7156)	161179	(26763 – 252927)
50% less red & processed meat**	261849	(35973 – 401549)	19560	(4351 – 32617)	20237	(8971 – 28696)	8827	(3757 – 13905)	310473	(53052 – 476767)
No red or processed meat	483539	(71147 – 708489)	38285	(8661 – 62908)	37033	(17228 – 50554)	16985	(7376 – 26204)	575842	(104412 – 848155)
**Replacing red & processed meat with vegetables and legumes**										
25% less red & processed meat	181455	(39805 – 276035)	4955	(1088 – 8356)	10573	(4571 – 15300)	4493	(1893 – 7156)	201476	(47356 – 306846)
50% less red & processed meat	345195	(79129 – 509819)	9915	(2187 – 16657)	20237	(8971 – 28696)	8827	(3757 – 13905)	384174	(94044 – 569078)
No red or processed meat***	620334	(154325 – 865894)	19560	(4351 – 32617)	37033	(17228 – 50554)	16985	(7376 – 26204)	693912	(183280 – 975269)
**Replacing milk consumption with plant-based drink#**										
25% less milk intake*	0	(0 – 0)	0	(0 – 0)	0	(0 – 0)	-2519	(-970 – -4490)	-2519	(-970 – -4490)
50% less milk intake**	0	(0 – 0)	0	(0 – 0)	0	(0 – 0)	-5089	(-1948 – -9139)	-5089	(-1948 – -9139)
No milk intake***	0	(0 – 0)	0	(0 – 0)	0	(0 – 0)	-10376	(-3923 – -18926)	-10376	(-3923 – -18926)
**Replacing SSB consumption with water#**										
25% less SSB intake*	24330	(-5680 – 47663)	0	(0 – 0)	1804	(916 – 2647)	0	(0 – 0)	26134	(-4764 – 50310)
50% less SSB intake**	48334	(-11378 – 94070)	0	(0 – 0)	3581	(1826 – 5238)	0	(0 – 0)	51915	(-9552 – 99308)
No SSB intake***	95248	(-22795 – 182980)	0	(0 – 0)	7048	(3619 – 10239)	0	(0 – 0)	102297	(-19176 – 193220)
* Combination "Minor changes" (sum)	128707	(-41 – 209812)	0	(0 – 0)	12376	(5487 – 17947)	1974	(922 – 2666)	143058	(6368 – 230425)
** Combination "Moderate changes" (sum)	310183	(24595 – 495619)	19560	(4351 – 32617)	23818	(10797 – 33934)	3738	(1809 – 4767)	357298	(41552 – 566936)
*** Combination "Extensive changes" (sum)	715583	(131530 – 1048874)	19560	(4351 – 32617)	44081	(20847 – 60794)	6609	(3452 – 7278)	785832	(160181 – 1149562)

SSB, sugar-sweetened beverages.

Replacements were of equal weight. Vegetables and legumes were 50:50. Numbers rounded to nearest 100.

#Replacement food category was neutral in the health impact model.

Table 1 (continued) Cumulative gains in YLL over 20 or 30 years, per sex and disease outcome

	Ischaemic heart disease		Stroke		Diabetes type 2		Colorectal cancer		Sum	
	**Estimate**	**Lower-Upper**	**Estimate**	**Lower-Upper**	**Estimate**	**Lower-Upper**	**Estimate**	**Lower-Upper**	**Estimate**	**Lower-Upper**
Men, cumulative gains in YLL over 20 years										
**Replacing red & processed meat with poultry/fish#**										
25% less red & processed meat*	111583	(6166 – 170983)	0	(0 – 0)	10334	(4588 – 14715)	930	(399 – 1466)	122847	(11153 – 187163)
50% less red & processed meat	211641	(12297 – 314688)	0	(0 – 0)	19228	(8892 – 26503)	1807	(789 – 2799)	232676	(21978 – 343989)
No red or processed meat	380577	(24401 – 534512)	0	(0 – 0)	33374	(16676 – 43384)	3401	(1535 – 5093)	417352	(42613 – 582989)
**Replacing red & processed meat with vegetables**										
25% less red & processed meat	143405	(19290 – 220936)	6239	(1384 – 10427)	10334	(4588 – 14715)	930	(399 – 1466)	160908	(25662 – 247544)
50% less red & processed meat**	267691	(38241 – 396036)	12251	(2758 – 20219)	19228	(8892 – 26503)	1807	(789 – 2799)	300978	(50680 – 445557)
No red or processed meat	467108	(75090 – 641209)	23626	(5471 – 38046)	33374	(16676 – 43385)	3401	(1535 – 5093)	527509	(98772 – 727732)
**Replacing red & processed meat with vegetables and legumes**										
25% less red & processed meat	188304	(42158 – 280044)	3148	(694 – 5295)	10334	(4588 – 14715)	930	(399 – 1466)	202716	(47839 – 301520)
50% less red & processed meat	343449	(82688 – 485920)	6239	(1384 – 10427)	19228	(8892 – 26503)	1807	(789 – 2799)	370723	(93753 – 525648)
No red or processed meat***	574058	(159024 – 743335)	12251	(2758 – 20219)	33374	(16676 – 43384)	3401	(1535 – 5093)	623085	(179993 – 812032)
**Replacing milk consumption with plant-based drink#**										
25% less milk intake*	0	(0 – 0)	0	(0 – 0)	0	(0 – 0)	-415	(-160 – -742)	-415	(-160 – -742)
50% less milk intake**	0	(0 – 0)	0	(0 – 0)	0	(0 – 0)	-841	(-321 – -1518)	-841	(-321 – -1518)
No milk intake***	0	(0 – 0)	0	(0 – 0)	0	(0 – 0)	-1727	(-648 – -3181)	-1727	(-648 – -3181)
**Replacing SSB consumption with water#**										
25% less SSB intake*	23537	(-5523 – 45926)	0	(0 – 0)	1629	(829 – 2387)	0	(0 – 0)	25166	(-4694 – 48313)
50% less SSB intake**	46650	(-11094 – 90075)	0	(0 – 0)	3227	(1652 – 4704)	0	(0 – 0)	49877	(-9442 – 94779)
No SSB intake***	91225	(-22279 – 172519)	0	(0 – 0)	6308	(3263 – 9099)	0	(0 – 0)	97533	(-19015 – 181618)
* Combination “Minor changes” (sum)	135120	(643 – 216909)	0	(0 – 0)	11963	(5417 – 17102)	515	(240 – 724)	147597	(6300 – 234734)
** Combination “Moderate changes” (sum)	314341	(27147 – 486111)	12251	(2758 – 20219)	22456	(10543 – 31207)	966	(468 – 1281)	350014	(40917 – 538818)
*** Combination “Extensive changes” (sum)	665283	(136745 – 915854)	12251	(2758 – 20219)	39682	(19940 – 52484)	1675	(888 – 1912)	718891	(160330 – 990469)

SSB, sugar-sweetened beverages.

Replacements were of equal weight. Vegetables and legumes were 50:50. Numbers rounded to nearest 100.

#Replacement food category was neutral in the health impact model.

Table 1 (continued) Cumulative gains in YLL over 20 or 30 years, per sex and disease outcome

	**Ischaemic heart disease**		**Stroke**		**Diabetes type 2**		**Colorectal cancer**		**Sum**	
	**Estimate**	**Lower-Upper**	**Estimate**	**Lower-Upper**	**Estimate**	**Lower-Upper**	**Estimate**	**Lower-Upper**	**Estimate**	**Lower-Upper**
Men, cumulative gains in YLL over 30 years										
**Replacing red & processed meat with poultry/fish#**										
25% less red & processed meat*	249711	(13762 – 383236)	0	(0 – 0)	23090	(10250 – 32882)	8186	(3515 – 12904)	280987	(27526 – 429022)
50% less red & processed meat	474874	(27450 – 708027)	0	(0 – 0)	42972	(19867 – 59239)	15912	(6945 – 24651)	533759	(54262 – 791917)
No red or processed meat	857798	(54485 – 1209869)	0	(0 – 0)	74610	(37266 – 97012)	29966	(13519 – 44890)	962374	(105270 – 1351772)
**Replacing red & processed meat with vegetables**										
25% less red & processed meat	321191	(43068 – 495852)	13625	(3023 – 22774)	23090	(10250 – 32882)	8186	(3515 – 12904)	366092	(59855 – 564412)
50% less red & processed meat**	601532	(85418 – 893018)	26760	(6021 – 44178)	42972	(19867 – 59239)	15912	(6945 – 24651)	687176	(118251 – 1021086)
No red or processed meat	1055329	(167885 – 1455739)	51627	(11947 – 83185)	74610	(37266 – 97012)	29966	(13519 – 44890)	1211532	(230617 – 1680827)
**Replacing red & processed meat with vegetables and legumes**										
25% less red & processed meat	422251	(94177 – 629497)	6875	(1514 – 11563)	23090	(10250 – 32882)	8186	(3515 – 12904)	460402	(109456 – 686846)
50% less red & processed meat	773336	(184909 – 1098399)	13625	(3023 – 22774)	42972	(19867 – 59239)	15912	(6945 – 24651)	845845	(214743 – 1205063)
No red or processed meat***	1300821	(356321 – 1692539)	26760	(6021 – 44178)	74610	(37266 – 97012)	29966	(13519 – 44890)	1432157	(413127 – 1878620)
**Replacing milk consumption with plant-based drink#**										
25% less milk intake*	0	(0 – 0)	0	(0 – 0)	0	(0 – 0)	-3655	(-1405 – -6529)	-3655	(-1405 – -6529)
50% less milk intake**	0	(0 – 0)	0	(0 – 0)	0	(0 – 0)	-7399	(-2822 – -13349)	-7399	(-2822 – -13349)
No milk intake***	0	(0 – 0)	0	(0 – 0)	0	(0 – 0)	-15188	(-5700 – -27966)	-15188	(-5700 – -27966)
**Replacing SSB consumption with water#**										
25% less SSB intake*	52553	(-12322 – 102604)	0	(0 – 0)	3639	(1852 – 5331)	0	(0 – 0)	56192	(-10471 – 107936)
50% less SSB intake**	104223	(-24748 – 201466)	0	(0 – 0)	7210	(3690 – 10509)	0	(0 – 0)	111433	(-21059 – 211975)
No SSB intake***	204045	(-49687 – 386695)	0	(0 – 0)	14093	(7290 – 20331)	0	(0 – 0)	218137	(-42396 – 407026)
* Combination “Minor changes” (sum)	302265	(1439 – 485840)	0	(0 – 0)	26729	(12102 – 38213)	4531	(2110 – 6375)	333524	(15650 – 530429)
** Combination “Moderate changes” (sum)	705755	(60669 – 1094484)	26760	(6021 – 44178)	50182	(23557 – 69748)	8514	(4123 – 11302)	791210	(94371 – 1219712)
*** Combination “Extensive changes” (sum)	1504865	(306634 – 2079234)	26760	(6021 – 44178)	88703	(44557 – 117343)	14778	(7819 – 16924)	1635106	(365031 – 2257679)

SSB, sugar-sweetened beverages.

Replacements were of equal weight. Vegetables and legumes were 50:50. Numbers rounded to nearest 100.

#Replacement food category was neutral in the health impact model.
